# Rapamycin Inhibits IGF-1-Mediated Up-Regulation of MDM2 and Sensitizes Cancer Cells to Chemotherapy

**DOI:** 10.1371/journal.pone.0063179

**Published:** 2013-04-30

**Authors:** Wei Du, Yong Yi, Haibo Zhang, Johann Bergholz, Junfeng Wu, Haoqiang Ying, Yujun Zhang, Zhi-Xiong Jim Xiao

**Affiliations:** 1 Department of Biochemistry, Boston University School of Medicine, Boston, Massachusetts, United States of America; 2 Center of Growth, Metabolism and Aging, College of Life Sciences and State Key Laboratory of Biotherapy, Sichuan University, Chengdu, China; 3 The University of Texas MD Anderson Cancer Center, Houston, Texas, United States of America; University of Texas Health Science Center at San Antonio/Greehey CCRI, United States of America

## Abstract

The Murine Double Minute 2 (MDM2) protein is a key regulator of cell proliferation and apoptosis that acts primarily by inhibiting the p53 tumor suppressor. Similarly, the PI3-Kinase (PI3K)/AKT pathway is critical for growth factor-mediated cell survival. Additionally, it has been reported that AKT can directly phosphorylate and activate MDM2. In this study, we show that IGF-1 up-regulates MDM2 protein levels in a PI3K/AKT-dependent manner. Inhibition of mTOR by rapamycin or expression of a dominant negative eukaryotic initiation factor 4E binding protein 1 (4EBP1) mutant protein, as well as ablation of eukaryotic initiation factor 4E (eIF4E), efficiently abolishes IGF-1-mediated up-regulation of MDM2. In addition, we show that rapamycin effectively inhibits MDM2 expression and sensitizes cancer cells to chemotherapy. Taken together, this study reveals a novel mechanism by which IGF-1 activates MDM2 via the mTOR pathway, and that pharmacologic inhibition of mTOR combined with chemotherapy may be more effective in treatment of a subset of cancers harboring increased MDM2 activation.

## Introduction

Abnormal activation of Murine Double Minute 2 (MDM2) has been established as an important causative factor in human cancer development. MDM2 functions as an ubiquitin E3 ligase to facilitate degradation of p53, a key regulator for cell proliferation, apoptosis and senescence in response to cellular stresses, such as DNA damage and oncogenic stress [1]. Amplification of the *MDM2* gene has been observed in a variety of human tumors and cancers, including soft tissue tumors, osteosarcoma, and esophageal carcinoma [2]. Notably, MDM2 has been shown to possess p53-independent oncogenic functions [3], [4]. Work from us and others have shown that MDM2 can target and inhibit retinoblastoma protein (Rb) via proteasome-mediated degradation [5], [6], [7], [8], [9]. MDM2 has also been shown to complex with and regulate protein stability and/or activity of a subset of proteins involved in cell proliferation and cell death, including p73 [10], [11], E2F1 [12], cyclin-dependent kinase inhibitor p21 [13], beta-arrestin, and G-protein-coupled receptor kinase 2 (GRK2) [14], [15].

It has been shown that MDM2 protein subcellular localization and functions are modulated by the PI3-Kinase (PI3K)/AKT pathway. AKT can directly phosphorylate MDM2 at Ser166 and Ser188, thus facilitating nuclear translocation [16] and p53 degradation, as well as p300 interaction [17], [18]. In addition, overexpression of AKT has been shown to stabilize MDM2 protein [19]. Recently, AKT has emerged as a critical regulator of mammalian target of rapamycin complex 1 (mTORC1). AKT inhibits the TSC2/TSC1 complex, leading to activation of mTORC1 [20]. Importantly, emerging evidence suggests that mTORC1 activity is critical for AKT oncogenic function. Indeed, accelerated tumor growth upon constitutive activation of AKT is reversed by inhibition of mTOR [21]. Similarly, mice expressing human AKT1 in the prostate develop a neoplastic phenotype, which is completely abolished by inhibition of mTOR [22]. Moreover, inactivation of AKT leads to inhibition of cell proliferation, which is dependent on mTORC1 [23]. These studies suggest that the AKT-mTOR pathway is crucial for tumor cell growth.

In this study, we show that insulin-like growth factor 1 (IGF-1) up-regulates MDM2 expression through the AKT-mTOR pathway. Inhibition of mTOR by rapamycin, expression of a dominant negative eukaryotic initiation factor 4E binding protein 1 (4EBP1) mutant or silencing of eukaryotic initiation factor 4E (eIF4E) efficiently abrogate IGF-1-mediated up-regulation of MDM2. In addition, we show that rapamycin effectively inhibits MDM2 expression and sensitizes cancer cells to chemotherapeutic drug-induced apoptosis.

## Results

### IGF-1 Induces MDM2 Expression in a PI3K-dependent Manner and does not Alter MDM2 Protein Stability or Steady-state mRNA Levels

**Figure 1:**
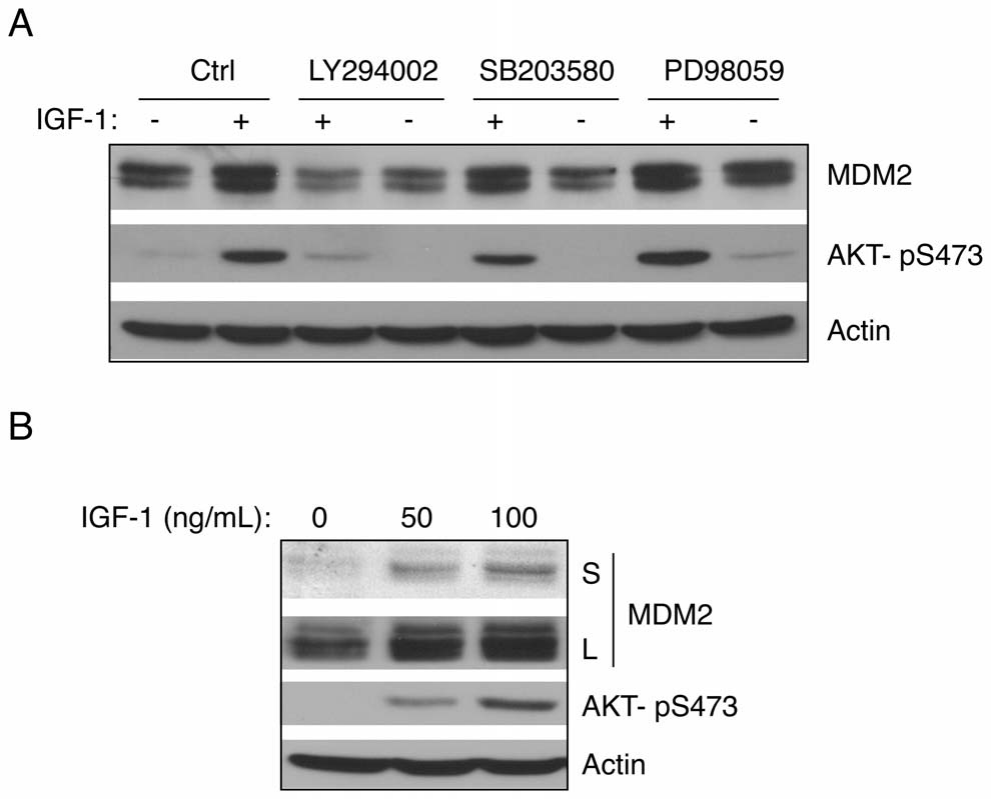
IGF-1 induces MDM2 expression in a PI3-Kinase-dependent pathway. (A) U2-OS cells were serum-starved for 24 hours and then pre-treated with either PI3-kinase (PI3K) inhibitor LY294002 (20 µM), MAP kinase kinase (MEK) inhibitor PD98059 (25 µM), or p38 stress-activated protein kinase inhibitor SB202190 (10 µM) for four hours, followed by treatment with IGF-1 (50 ng/mL) for six hours. Cells were lysed and subjected to western blot analysis. (B) H1299 cells were serum-starved by incubating in the absence of FBS for 24 hours and treated with IGF-1 for six hours as indicated. Cell lysates were subjected to western blot analysis. S: short exposure; L: long exposure.

We have previously shown that IGF-1 modulates the cyclin-dependent kinase inhibitor p21 to impact on cell survival upon genotoxic stress [24]. Since IGF-1 is known to activate the PI3K/AKT pathway, we were interested in deciphering the role of PI3K/AKT in IGF-1-mediated cell survival. As shown in Figure 1A, IGF-1 treatment of serum-starved human osteosarcoma U2-OS cells (p53 wild type) clearly led to AKT activation, as shown by an increase in AKT phosphorylation. Notably, IGF-1 induced MDM2 protein expression, as shown by an increase of both MDM2 protein bands detected by an MDM2-specific antibody, SMP14, consistent with previous reports [6], [9], [25]. This effect of IGF-1 on MDM2 was effectively blocked by treatment with LY294002, a selective PI3K inhibitor [26], but not by PD98059, an inhibitor of MAP kinase kinase (MEK), or by SB203580, a specific inhibitor of p38 stress-activated protein kinase [27]. These data suggest that IGF-1 up-regulates MDM2 protein expression through the PI3K-AKT signaling cascade.

Since we have previously shown that IGF-1 can activate p53, and MDM2 is a direct downstream p53 target [24], we asked whether the effect of IGF-1 on the expression of MDM2 is dependent on p53. We treated p53-null human non-small cell lung carcinoma H1299 cells with IGF-1. IGF-1 was still able to induce MDM2 protein expression in H1299 cells in a dose-dependent manner (Figure 1B), indicating that IGF-1-mediated up-regulation of MDM2 is independent of p53.

**Figure 2:**
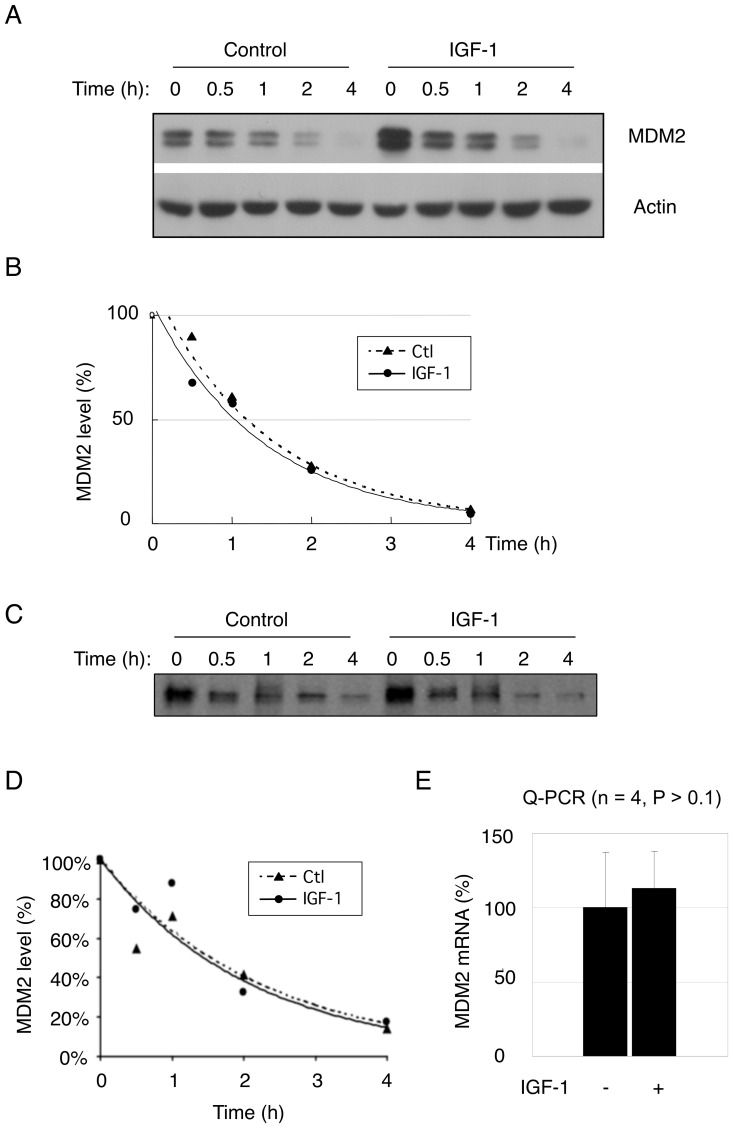
IGF-1 does not significantly alter MDM2 protein stability or steady state mRNA levels. (A) U2-OS cells were serum-starved for 24 hours prior to treating with IGF-1 (50 ng/mL) for six hours. Cells were then treated with 50 µg/mL cycloheximide (CHX) in the presence or absence of IGF-1 and collected at the indicated times. Cell lysates were subjected to western blot analysis for MDM2 and actin. (B) Quantitative analyses of MDM2 protein levels from cells treated with CHX were performed by densitometry scanning for each individual MDM2 protein band and the corresponding actin protein band. The ratio of MDM2 over actin at the zero time point was arbitrarily set as 100 percent. Three independent experiments were performed with similar outcomes. (C) U2-OS cells were treated with IGF-1 (50 ng/mL) for six hours and metabolically labeled with ^35^S-methionine for forty minutes, and then chased with cold methionine. Cell lysates with equal amounts of radioactivity were immunoprecipitated with SMP14 antibody against MDM2. Proteins were separated by SDS-PAGE and visualized by autoradiography. (D) Quantitative analysis was performed by densitometry scanning using ImageQuant software. (E) Serum-starved U2-OS cells were treated with IGF-1 (50 ng/mL) for six hours. MDM2 gene expression levels were determined by quantitative-PCR (Q-PCR) and normalized to GAPDH expression. Data presented as means and SE of two independent experiments performed in duplicate.

Next, we investigated whether IGF-1 up-regulates MDM2 protein levels by increasing its protein stability. As expected, IGF-1 increased MDM2 protein expression (Figure 2A). However, IGF-1 treatment did not significantly alter MDM2 protein half-life, as determined by treatment with the protein biosynthesis inhibitor cycloheximide (Figure 2A and 2B) and by pulse-chase experiments (Figure 2C and 2D). We then examined whether IGF-1 induces MDM2 by increasing its mRNA levels. However, quantitative PCR (Q-PCR) analysis showed that MDM2 mRNA levels were not significantly affected by IGF-1 treatment (Figure 2E), indicating that IGF-1 does not affect steady-state mRNA levels.

### Inhibition of mTOR by Rapamycin Blocks IGF-1- and Heregulin-mediated Up-regulation of MDM2

**Figure 3:**
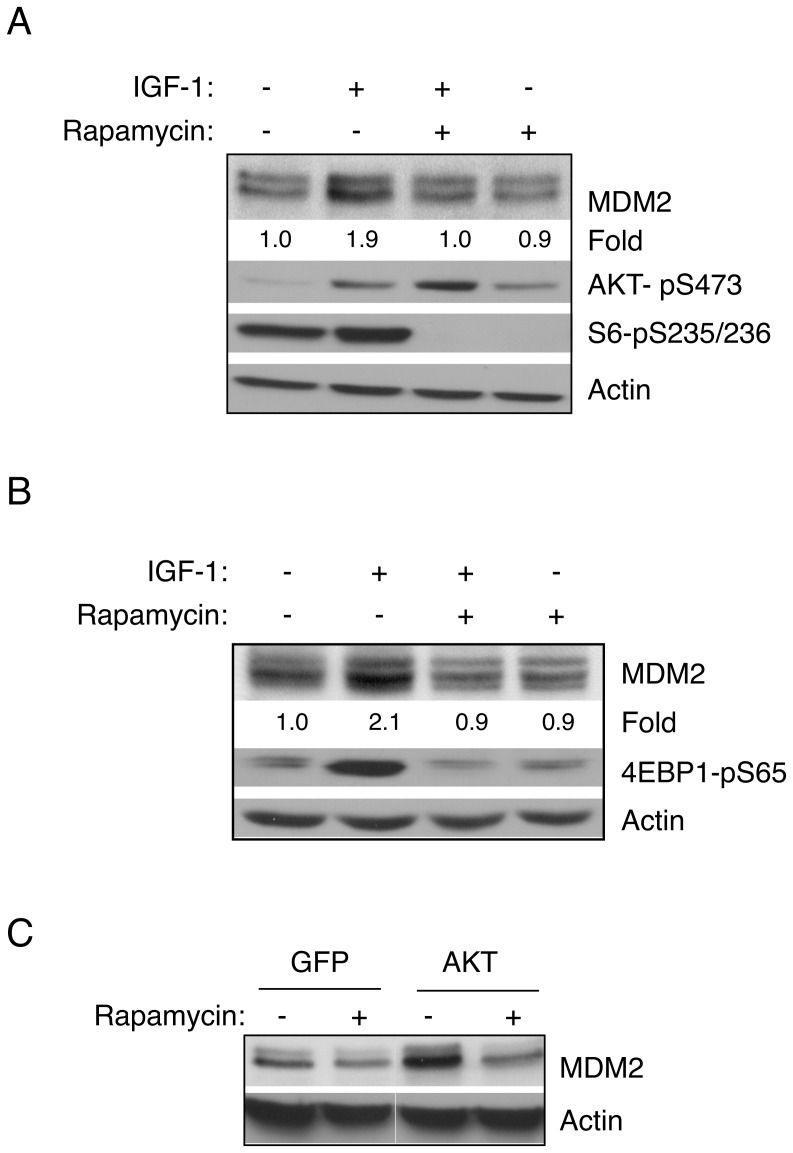
Inhibition of mTOR by rapamycin blocks IGF-1 mediated up-regulation of MDM2. U2-OS (A) or H1299 (B) cells were serum-starved and pre-treated with rapamycin (50 nM) for four hours, and then treated with IGF-1 (50 ng/mL) for six hours. Cells were lysed and subjected to western blot analysis. Quantitative analyses of MDM2 protein levels were performed by densitometry scanning for each individual MDM2 protein band and the corresponding actin protein band. The ratio of MDM2 over actin in the control sample was arbitrarily set as 1.0. (C) U2-OS cells were infected with recombinant adenovirus encoding GFP or myristic-AKT for 24 hours before treatment with rapamycin (100 nM) for four hours. Cell lysates were subjected to western blot analysis.

AKT has been shown to be an important regulator of mTORC1 activity. Thus, we investigated whether the mTOR pathway plays a role in IGF-1-mediated up-regulation of MDM2. To this end, we serum-starved and then pretreated U2-OS cells with rapamycin, a selective inhibitor for mTOR [28], prior to IGF-1 treatment. As expected, rapamycin blocked phosphorylation of the ribosomal protein S6 (Figure 3A). Notably, rapamycin also blocked IGF-1-induced MDM2 up-regulation (Figure 3A). Similar results were observed in H1299 cells, in which rapamycin abrogated IGF-1-induced MDM2 up-regulation and phosphorylation of 4EBP1 (Figure 3B). Moreover, expression of constitutively active AKT (myristic-AKT) led to an increase of MDM2 protein levels, which was completely blocked in the presence of rapamycin (Figure 3C). Taken together, these data strongly support the notion that a rapamycin-sensitive pathway is critical for up-regulation of MDM2 via IGF-1/PI3K/AKT signaling.

**Figure 4:**
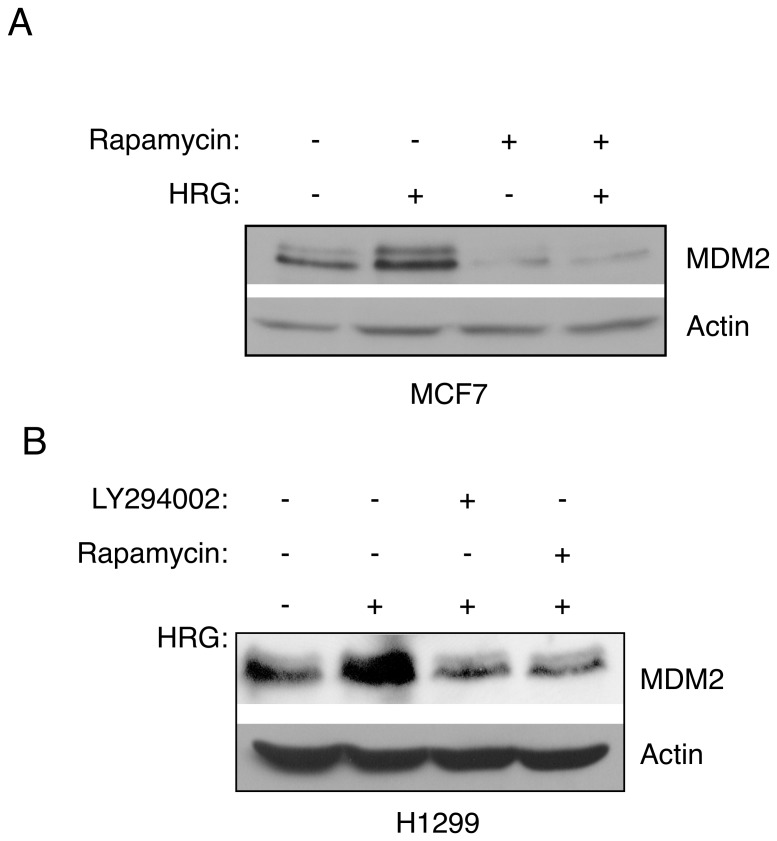
Inhibition of mTOR by rapamycin blocks Her2-mediated up-regulation of MDM2. (A) MCF-7 cells were serum-starved and pre-treated with rapamycin (50 nM) for four hours, and then treated with Heregulin-beta (HRG; 25 ng/mL) for 24 hours. Cells were lysed and subjected to western blot analysis. (B) Serum-starved H1299 cells were pre-treated with rapamycin (50 nM) or LY294002 (20 µM) for four hours, and then treated with Heregulin-beta (25 ng/mL) for 24 hours. Cell lysates were subjected to western blot analysis.

Since Heregulin can activate AKT, we asked whether this ligand could up-regulate MDM2 in an mTOR-dependent manner. MCF-7 cells or H1299 cells were serum-starved prior to treatment with rapamycin, and then treated with heregulin-beta (HRG). As shown on Figure 4A, activation of AKT signaling by HRG effectively induced MDM2 protein expression, which was completely blocked by rapamycin in both MCF-7 and H1299 cells (Figure 4A and 4B). In addition, the PI3K inhibitor LY294002 effectively inhibited the effect of HRG on the up-regulation of MDM2 (Figure 4B), indicating that Heregulin-mediated up-regulation of MDM2 is dependent on PI3K. Together, these data indicate that a rapamycin-sensitive pathway is required for up-regulation of MDM2 by the IGF-1 or Heregulin signaling pathways.

### Expression of a Mutant 4EBP1 Defective in mTOR Phosphorylation Attenuates IGF-1-mediated Up-regulation of MDM2

**Figure 5:**
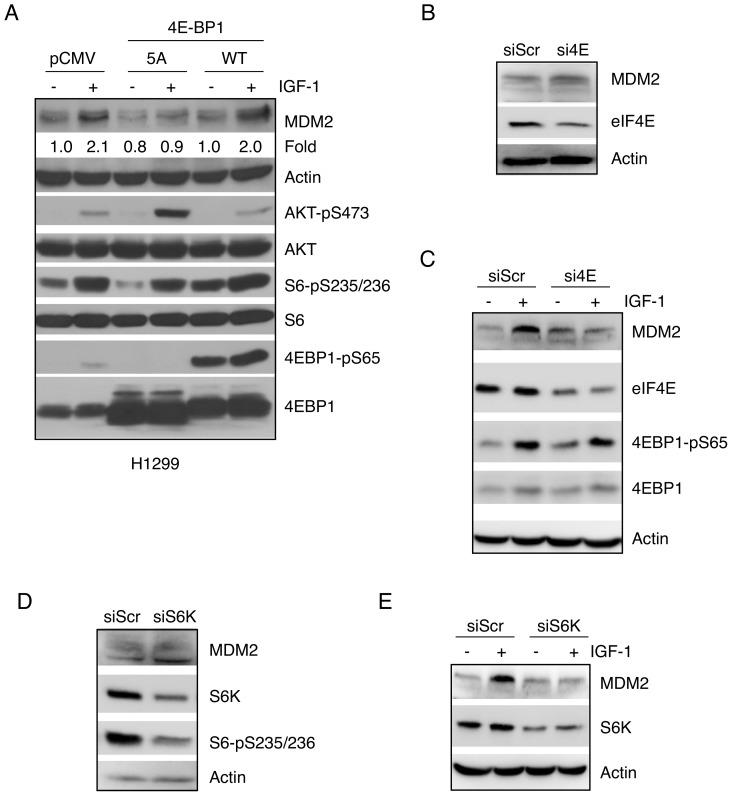
Signaling downstream of mTOR is required for IGF-1-induced MDM2 up-regulation. (A) H1299 cells were transiently transfected with wild-type 4EBP1, 4EBP1-5A, or vector plasmid. Twenty-four hours after transfection, cells were split and maintained for an additional 24 hours prior to serum starvation for 24 hours. Cells were then treated with or without IGF-1 (50 ng/mL) for six hours. Cells were lysed and subjected to western blotting. Quantitative analyses of MDM2 protein levels were performed by densitometry scanning for each individual MDM2 protein band and the corresponding actin protein band. The ratio of MDM2 over actin in the control sample (lane 1) was arbitrarily set as 1.0. (B) U2-OS cells were transiently transfected with siRNA specific against eIF4E (si4E) or a scrambled control (siScr). Cell lysates were subjected to western blotting, as shown. (C) U2-OS cells transfected with si4E or siScr were serum-starved for 24 hours prior to treatment with or without 5 ng/mL IGF-1 for six hours. Cell lysates were subjected to western blotting, as shown. (D) U2-OS cells were transiently transfected with siRNA specific against S6K (siS6K) or a scrambled siRNA (siScr). Cell lysates were subjected to western blotting, as shown. (E) U2-OS cells transfected with siS6K or siScr were serum-starved for 24 hours prior to treatment with or without 5 ng/mL IGF-1 for six hours. Cell lysates were subjected to western blotting, as shown.

We next determined whether eIF4E, a major downstream target of mTOR, is involved in IGF-1-mediated up-regulation of MDM2. Since mTOR phosphorylates 4EBP1, resulting in its dissociation from eIF4E and leading to the formation of the translation initiation complex [29], we used mutant 4EBP1-5A, which lacks the five Ser/Thr phosphorylation sites targeted by mTOR and thus acts as a dominant negative inhibitor by binding elF4E to prevent translation initiation [30]. To this end, H1299 cells were transiently transfected with either wild type 4EBP1 or 4EBP1-5A, followed by serum starvation and IGF-1 treatment. As shown in Figure 5A, IGF-1 treatment led to a sharp increase in phosphorylation of AKT, S6 and 4EBP1, correlated with a marked increase in MDM2 expression. Notably, expression of wild-type 4EBP1 did not significantly alter the effect of IGF-1 on MDM2 expression, likely due to effective phosphorylation of 4EBP1 in H1299 cells. However, IGF-1-mediated MDM2 up-regulation was significantly inhibited by ectopic expression of 4EBP1-5A. Next, in order to investigate the role of eIF4E directly, we ablated endogenous eIF4E using small interfering RNA (siRNA) in U2-OS cells. While silencing eIF4E did not significantly affect basal MDM2 expression (Figure 5B), knockdown of eIF4E completely abrogated the effect of IGF-1 on increasing MDM2 levels, despite evident 4EBP1 phosphorylation (Figure 5C).

In addition to phosphorylating and inhibiting 4EBP1, mTOR phosphorylates and activates ribosomal protein S6 Kinase (S6K), which in turn phosphorylates and activates ribosomal protein S6 in order to enhance protein translation. Thus, we ablated S6K by siRNA in U2-OS cells. As shown in Figure 5D and 5E, we observed similar effects as eIF4E knockdown, indicating IGF-1-mediated MDM2 up-regulation requires mTOR signaling, which involves both the eIF4E and S6K pathways.

### Rapamycin Treatment Reduces MDM2 Expression and Sensitizes Cancer Cells to Doxorubicin- Induced Apoptosis

**Figure 6:**
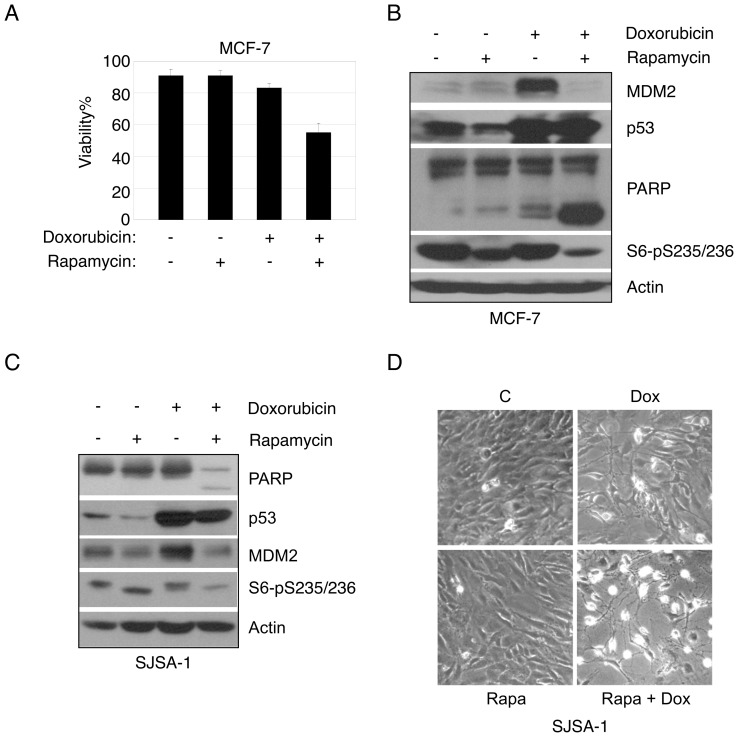
Rapamycin treatment reduces MDM2 expression and sensitizes cancer cells to doxorubicin-induced apoptosis. (A–B) MCF-7 cells were maintained in normal growth media, pretreated with rapamycin (100 nM) for 48 hours, and then treated with doxorubicin (0.6 µg/mL) for 24 hours. Both floating and adherent cells were collected, and subjected to trypan blue exclusion assay (A) and western blot analysis (B). Cell viability is presented as a percentage of live cells over total cells counted. Data presented as means and SE of three independent experiments performed in triplicate. (C) SJSA-1 cells were maintained in normal growth media, pretreated with rapamycin (100 nM) for 48 hours and then treated with doxorubicin (1 µg/mL) for 36 hours. Both floating and adherent cells were collected, and subjected to western blot analysis. (D) Morphology of SJSA-1 cells was recorded by phase contrast microscopy (100×).

Since MDM2 is a key negative regulator of p53, which is essential for DNA damage-induced apoptosis, we investigated the effect of rapamycin on doxorubicin-induced apoptosis in p53-positive cancer cells. We chose MCF-7 breast cancer cells because they harbor a somatic mutation in the PIK3CA gene that confers a constitutively active AKT-mTOR pathway [31]. MCF-7 cells were pretreated with rapamycin, followed by treatment with a low dose of doxorubicin. Cell viability was assessed by trypan blue exclusion assay and western blot analysis for cleavage of poly ADP-ribose polymerase (PARP), a biochemical marker for apoptosis. Under these conditions, neither rapamycin nor doxorubicin alone significantly induced apoptosis. However, in the presence of rapamycin, doxorubicin effectively induced cell death (Figure 6A). Notably, rapamycin completely blocked up-regulation of MDM2, but not of p53 (Figure 6B). Similar phenomena were observed in human osteosarcoma SJSA-1 cells, which harbor MDM2 amplification [25] (Figure 6C and 6D). Together, these data suggest that rapamycin suppresses MDM2 protein expression, resulting in sensitization of cancer cells to doxorubicin-induced cell death.

**Figure 7:**
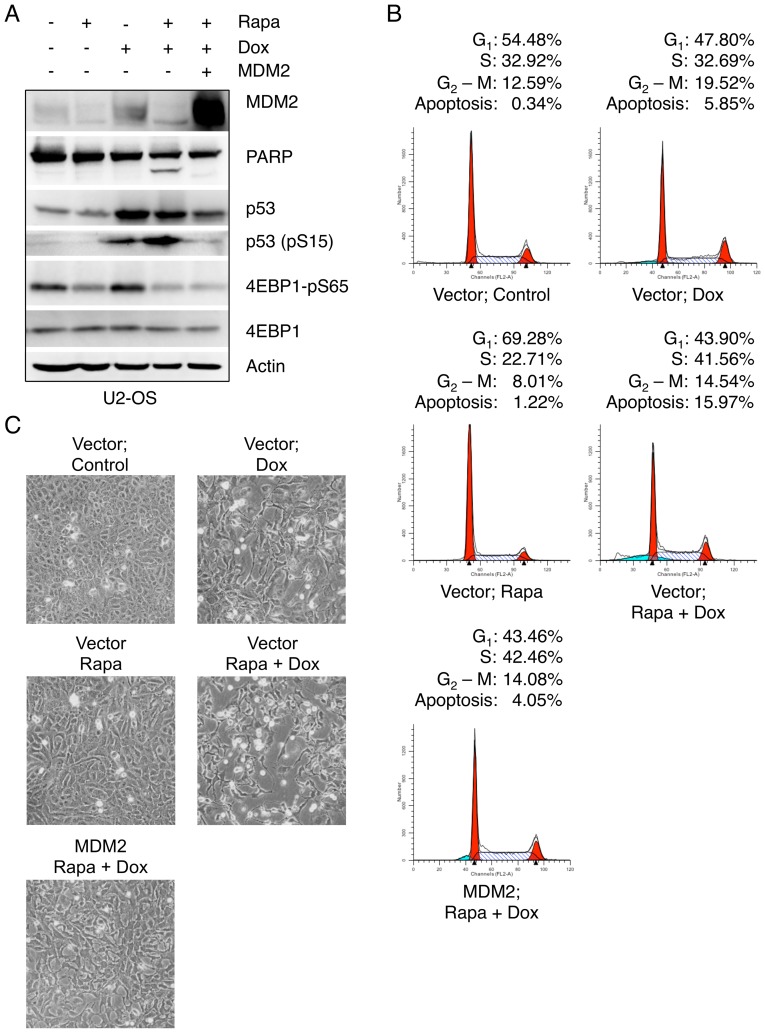
Ectopic MDM2 expression abrogates enhanced effect of combined rapamycin/doxorubicin therapy. (A–B) U2-OS cells were transiently transfected with MDM2 or a vector control. Twenty-four hours post transfections, cells were pretreated with rapamycin (100 nM) for 48 hours and then treated with doxorubicin (1 µg/mL) for 36 hours. Both floating and adherent cells were collected, and subjected to western blotting (A) or flow cytometry analysis (B). (C) Morphology of U2-OS cells was recorded by phase contrast microscopy (100×).

We next investigated whether down-regulation of MDM2 plays a causative role in sensitization of cells to doxorubicin by rapamycin treatment. We treated U2-OS cells with rapamycin and doxorubicin either in the presence or absence of ectopic expression of MDM2. As shown in Figure 7A, MDM2 expression dramatically reverted cleaved PARP levels, compared to cells expressing a vector control. Notably, MDM2 expression reduced p53 protein levels, as well as Ser15 phosphorylation on p53. Moreover, MDM2 expression effectively rescued cell death induced by rapamycin plus doxorubicin combination treatment (Figure 7B and 7C). These data indicate that inhibition of MDM2 up-regulation by rapamycin is largely responsible for sensitizing cells to chemotherapeutic treatment.

## Discussion

In this study, we demonstrated that IGF-1 up-regulates MDM2 protein levels via the mTOR pathway, thus adding another layer of fine regulation to the MDM2-p53 pathway by the IGF-1/AKT signaling cascade. AKT has been previously shown to physically interact with and phosphorylate MDM2, which facilitates MDM2 nuclear translocation [16]. Phosphorylation of MDM2 enhances MDM2’s interaction with p300, thus increasing ubiquitin E3 ligase activity toward p53 and facilitating p53 ubiquitination and degradation [17]. In addition, phosphorylation of MDM2 on Ser166 and Ser188 by AKT has been shown to stabilize MDM2 protein by inhibiting its self-ubiquitination [19]. However, it was also reported that AKT-mediated phosphorylation of MDM2 does not seem to dramatically affect MDM2 subcellular localization [18]. Thus, although it is clear that AKT positively regulates MDM2, the precise molecular mechanisms seem to be cell type- and context-dependent.

Previous studies suggest that MDM2 can be regulated at the translational level. Enhanced translation of MDM2 has been observed in human choriocarcinoma cell lines [32]. The MDM2 mRNA contains a distinct 5' untranslated region (UTR) that plays a role in translational efficiency [33]. In addition, increased MDM2 expression induced by BCR/ABL expression is associated with enhanced MDM2 translation due to regulation at the 5′ UTR of MDM2 mRNA [34]. Moreover, inhibition of IGF-1 signaling, either by knockout of IGF-1 receptor (IGF-1R) or by an IGF-1R kinase inhibitor, has been shown to decrease MDM2 translation mediated via its 5′ UTR [35]. Notably, it has been reported that hepatocyte growth factor (HGF) facilitates MDM2 translation via mTOR in embryonic hepatocytes [36].

In this study, we show that both IGF-1 and heregulin signaling activate MDM2 through the AKT-mTOR pathway. This study has two important implications. First, cellular responses to a variety of stresses converge on the p53-MDM2 pathway, which serves as the central sensor for stress. Second, there are two major regulatory mechanisms to modulate MDM2 protein levels: p53-dependent transcriptional regulation and mTOR-dependent translational control. Stress signals, including DNA damage, hypoxia, nutrient deprivation and oxidative stress, activate p53, which in turn transcriptionally up-regulates MDM2 expression. Growth factor signaling, on the other hand, activates the AKT-mTOR pathway to enhance MDM2 protein translation. This delicate regulation of MDM2 ensures that p53 is under tight control. Moreover, our data show that eIF4E or S6K ablation do not significantly affect MDM2 basal levels. However, silencing of either eIF4E or S6K completely abolished IGF-1-induced MDM2 up-regulation. These results indicate that the mTOR pathway is required for growth factor-mediated MDM2 up-regulation.

Inhibition of mTOR using specific pharmacological inhibitors results in G1/S cell cycle arrest, attenuation of cell growth, and apoptosis [37]. Pre-clinical studies indicate that rapamycin and its derivatives inhibit cancer cell growth and proliferation, which has been shown both *in vitro* and in a variety of human cancers using animal models [38]. In addition, it was reported that inhibition of mTOR promotes apoptosis through translational control of Mcl-1, a bcl-2 like family member [39]. However, in most cases, inhibition of mTOR alone does not effectively induce apoptosis; rather, it sensitizes cells to apoptotic stimuli. For instance, rapamycin increases the ability of cisplatin to induce apoptosis in human promyelocytic leukemia cell lines and ovarian cancer cell lines [40]. RAD001, a rapamycin derivative, has been shown to sensitize a number of p53-positive cancer cells to cisplatin-induced apoptosis by inhibiting p21 protein expression through the repression of general translation [41]. Moreover, rapamycin sensitized multiple myeloma cell lines to dexamethasone-induced apoptosis, associated with decreased expression of cyclin D2 and survivin [42]. It has also been reported that expression of constitutively active mutant 4E-BP1 mimics the effect of rapamycin in enhancing apoptosis in multiple myeloma cells, suggesting that the mTOR inhibitors sensitize cells by inhibiting cap-dependent translation [43]. Consistent with this notion, small molecules that target cap-dependent translation initiation by inhibition of eIF4A can restore drug sensitivity in a mouse lymphoma model [44].

In this study, we observed that rapamycin and doxorubicin combination treatment led to reduced MDM2 expression, which resulted in increased apoptosis, implicating that p53 may be involved in this process. Our data showed that doxorubicin up-regulated p53, as expected. However, while rapamycin/doxorubicin combination treatment did not significantly affect p53 protein levels, phosphorylation at serine 15 on p53 were increased, suggesting that p53 activity was induced. Importantly, ectopic expression of MDM2 reversed apoptosis, concomitant with reduced p53 protein levels and serine 15 phosphorylation. Nonetheless, MDM2 may also have p53-independent functions to inhibit apoptosis in the presence of doxorubicin/rapamycin combination treatment.

In summary, our study shows that rapamycin sensitizes cancer cells to chemotherapeutic drug-induced apoptosis and suggests that mTOR-targeted anticancer-therapy combined with chemotherapy may be more effective in treatment of p53-positive cancers.

## Materials and Methods

### Cell Culture, Drug Treatments and Plasmids

Human breast carcinoma MCF-7 cells, osteosarcoma U2-OS cells and non-small cell lung carcinoma H1299 cells were maintained in Dulbecco’s modified Eagle medium (DMEM) containing 4.5 g/L glucose supplemented with 10% fetal bovine serum (FBS; certified, GIBCO), 100 units/mL penicillin (GIBCO), 100 µg/mL streptomycin (GIBCO), and 2 mM L-Glutamine (GIBCO). All cell lines were passaged using 0.05% Trypsin-EDTA solution (GIBCO). Cells were maintained in a humidified 37°C incubator under a 5% CO_2_ atmosphere. Cells were obtained from American Type Culture Collection (ATCC). MCF-7, U2-OS or H1299 cells at 60–70% confluence were serum starved for 24 hours in serum-free DMEM prior to IGF-1 treatment. IGF-1 (Sigma) was prepared as a 100 µg/mL stock solution in water. Chemical inhibitors LY294002, PD98059, SB202190 (Calbiochem), and Rapamycin (Sigma) were prepared as 1,000× stock solutions. Doxorubicin (Sigma) was prepared as a 2 mg/mL stock solution. Heregulin-beta (Upstate) was prepared as a 20 µg/mL solution in PBS. For UV treatment, media were removed and the cells irradiated with lids removed in a Spectrolinker XL-1000 UV Crosslinker (Spectronics Corporation). After irradiation, media were added to the dishes and the cells were grown as indicated for each experiment before collection of both floating and adherent cells.

### Adenoviral Infection, Transient Transfection and RNA Interference

Cells at 60% confluence were infected with Adenovirus encoding AKT or a vector control, and incubated for two hours at 37°C with mild agitation at twenty-minute intervals, followed by the addition of culture media and incubated at 37°C with 5% CO_2_ for 24 hours. H1299 cells at 80% confluency or U2-OS cells at 50% confluency were transfected using Lipofectamine 2000 transfection reagent (Invitrogen), according to manufacturer’s instructions. Media were replaced eight (H1299) or six (U2-OS) hours after transfection. Small interfering RNA against eIF4E (AAGCAAACCUGCGGCUGAUCU), S6K (GGACATGGCAGGAGTGTTT) and scrambled siRNA controls specific for each siRNA oligonucleotide were purchased from Shanghai GenePharma Co., Ltd. and transfected using Lipofectamine 2000 (Invitrogen).

### Western Blot Analysis

Cells were collected, washed with PBS, and resuspended in EBC_250_ lysis buffer (250 mM NaCl, 50 mM Tris pH 8.0, 0.5% Nonidet P-40, 50 mM NaF, 1 mM phenylmethylsulfonyl fluoride, 2 µg/mL aprotinin, and 2 µg/mL leupeptin). Protein concentration was determined using the Bio-Rad Protein Assay Reagent (Bio-Rad). Equal amounts of protein were loaded, separated by SDS-PAGE, transferred to PVDF membranes (Millipore), and hybridized to an appropriate primary antibody and HRP-conjugated secondary antibody for subsequent detection by enhanced chemiluminescence. Monoclonal antibody SMP14 specific for MDM2 (Santa Cruz) was used at 1∶200 dilutions. Monoclonal antibody DO-1 specific for p53 (Santa Cruz) was used at a dilution of 1∶250. Polyclonal antibody C-11 specific for actin (Santa Cruz) was used at a dilution of 1∶1000. Antibodies specific for PARP (#9542), phospho-AKT (Ser473; #9271), total AKT (#9272), Phospho-S6 Ribosomal Protein (serine 235/236; #2211), S6 Ribosomal Protein (#2217), S6 Kinase (#9202), eIF4E, Phospho-4EBP1 (Ser65; #9456) or 4EBP1 (#9452) were purchased from Cell Signaling Technology and used at 1∶1000 dilutions.

### Flow Cytometry Analysis

Cells were trypsinized, washed with cold PBS, and fixed in 70% ethanol at 4°C overnight. Cells were stained with propidium iodide (PI) supplemented with 80 µg/mL RNase A at 37°C in dark for one hour. Cells were then subjected to flow cytometry analysis by FACScan Flow Cytometer (Becton Dickson), and the data were analyzed using Cell Quest software (Becton Dickson).

### Quantitative PCR

Total RNA was extracted from cells using Tryzol according to manufacturer’s instructions (Gibco). RNA was reverse transcribed using iScript cDNA Synthesis Kit (BioRad). The reaction was also carried out in the absence of reverse transcriptase to serve as a negative control. Q-PCR was carried out in 25 µL reaction volumes (8 ng cDNA, 12.5 µL Taqman Universal PCR Master Mix, 1.25 µL Demand Gene Expression Reagent; Applied Biosystems) for both MDM2 and GAPDH. The Q-PCR reaction was performed in an ABI Prism 7000 Sequence detection System (Applied Biosystems). The Q-PCR conditions were as follows: 10 minutes at 95°C followed by 40 cycles of denaturing at 95°C for 15 seconds and annealing/extending at 60°C for one minute. Relative quantitation values were calculated using the ΔΔCt method.

### Cell Viability Assay

Both floating and adherent cells were collected and resuspended in PBS. Equal volumes of trypan blue stain solution (0.4%; Gibco) and cell suspension were mixed and stained for five minutes at room temperature. The numbers of stained and unstained cells were counted with a hemacytometer. At least 500 cells per sample were counted. The percentage of viability represents the ratio of unstained cells to total cells. Student’s *t-*test was used to determine statistical significance of differences between groups.
