# Correction: Rapamycin Inhibits IGF-1-Mediated Up-Regulation of MDM2 and Sensitizes Cancer Cells to Chemotherapy

**DOI:** 10.1371/annotation/f1a4d192-57b9-43fb-a248-379e0f5a46ca

**Published:** 2013-12-17

**Authors:** Wei Du, Yong Yi, Haibo Zhang, Johann Bergholz, Junfeng Wu, Haoqiang Ying, Yujun Zhang, Zhi-Xiong Jim Xiao

Haibo Zhang should be listed as an equal contributor and a co-first author with Wei Du and Yong Yi. 

